# Gene Expression Profiling in Mouse Embryonic Stem Cells Reveals Glycogen Synthase Kinase-3-Dependent Targets of Phosphatidylinositol 3-Kinase and Wnt/β-Catenin Signaling Pathways

**DOI:** 10.3389/fendo.2014.00133

**Published:** 2014-08-13

**Authors:** Colleen M. Bartman, Jennifer Egelston, Sravya Kattula, Leigh C. Zeidner, Anthony D’Ippolito, Bradley W. Doble, Christopher J. Phiel

**Affiliations:** ^1^Department of Integrative Biology, University of Colorado Denver, Denver, CO, USA; ^2^Center for Human and Molecular Genetics, Research Institute at Nationwide Children’s Hospital, Columbus, OH, USA; ^3^Stem Cell and Cancer Research Institute, Department of Biochemistry and Biomedical Sciences, McMaster University, Hamilton, ON, Canada

**Keywords:** embryonic stem cells, glycogen synthase kinase-3, Wnt, phosphatidylinositol 3-kinase, microarray, quantitative PCR, signal transduction, gene expression

## Abstract

Glycogen synthase kinase-3 (Gsk-3) activity is an important regulator of numerous signal transduction pathways. Gsk-3 activity is the sum of two largely redundant proteins, Gsk-3α and Gsk-3β, and in general, Gsk-3 is a negative regulator of cellular signaling. Genetic deletion of both *Gsk-3*α and *Gsk-3*β in mouse embryonic stem cells (ESCs) has previously been shown to lead to the constitutive activation of the Wnt/β-catenin signaling pathway. However, in addition to Wnt signaling, all Gsk-3-regulated pathways, such as insulin signaling, are also affected simultaneously in *Gsk-3*α^−^*^/^*^−^; *Gsk-3*β^−^*^/^*^−^ESCs. In an effort to better understand how specific signaling pathways contribute to the global pattern of gene expression in *Gsk-3*α^−^*^/^*^−^; *Gsk-3*β^−^*^/^*^−^ESCs, we compared the gene expression profiles in *Gsk-3*α^−^*^/^*^−^; *Gsk-3*β^−^*^/^*^−^ ESCs to mouse ESCs in which either Wnt/β-catenin signaling or phosphatidylinositol 3-kinase (PI3K)-dependent insulin signaling are constitutively active. Our results show that Wnt signaling has a greater effect on up-regulated genes in the *Gsk-3*α^−^*^/^*^−^; *Gsk-3*β^−^*^/^*^−^ESCs, whereas PI3K-dependent insulin signaling is more responsible for the down-regulation of genes in the same cells. These data show the importance of Gsk-3 activity on gene expression in mouse ESCs, and that these effects are due to the combined effects of multiple signaling pathways.

## Introduction

Glycogen synthase kinase-3 (Gsk-3) is an intracellular serine/threonine kinase activity that was originally identified as the rate-limiting step in the glycogen synthesis pathway (1). When the genes encoding Gsk-3 were cloned, Gsk-3 activity was revealed as the combined action of two distinct genes, *Gsk-3*α and *Gsk-3*β (2). Gsk-3 isoforms show a high degree of similarity at the amino acid level, including 98% similarity within the catalytic domain. Despite divergence at the amino- and carboxyl-termini of each isoform, it appears that the unstructured carboxyl-termini are essential for Gsk-3 activity (3). Rare among kinases, Gsk-3 is active at a basal state, while pathway activation from upstream signaling cascades results in the inhibition of Gsk-3 activity (4–6).

There are two primary mechanisms responsible for Gsk-3 inhibition – one that regulates the phosphorylation of Gsk-3α and Gsk-3β on serines 21 and 9, respectively, and another that is independent of this phosphorylation event. Several signaling pathways, including protein kinase A (PKA), Hedgehog, transforming growth factor-β (TGF-β), nuclear factor of activated T-cells (NF-AT), and phosphatidylinositol 3-kinase (PI3K)-dependent insulin signaling, all affect Gsk-3 activity via phosphorylation of the amino-terminal serines (4–6). Insulin binding to its receptor triggers the activation of PI3K, which phosphorylates and activates Akt, which in turn phosphorylates and inhibits Gsk-3 activity (7). We have previously shown that stable expression of a constitutively active form of the p110α catalytic subunit of PI3K (termed p110*) (8) in WT mouse ESCs can effectively lead to the phosphorylation of Gsk-3 (9), and expression of p110* has been shown to activate insulin signaling in mouse ESCs (10).

Gsk-3 activity also plays a central role in Wnt signaling as it is part of a cytosolic protein complex termed the β-catenin destruction complex (11). The role of Gsk-3 is to directly phosphorylate β-catenin (12, 13), which targets β-catenin for ubiquitin-mediated degradation (14), keeping the Wnt pathway inactive. Activation of the Wnt signaling pathway inhibits Gsk-3 activity, but this effect is not mediated through amino-terminal serine phosphorylation. Instead, upon Wnt ligand binding to a Frizzled/Lrp receptor complex, the destruction complex is neutralized via the translocation of Gsk-3 to the cell membrane, where it phosphorylates Lrp (15, 16). Gsk-3 is then subsequently endocytosed into multivesicular bodies, further insulating it from β-catenin (17). Once β-catenin protein levels accumulate to high levels in the cytoplasm, it is subsequently translocated to the nucleus where it can directly complex with lymphocyte enhancer factor (Lef)/T-cell factor (Tcf) proteins to activate Wnt target genes (18, 19). Gsk-3α and Gsk-3β are redundant with respect to their roles in regulating Wnt signaling; the genetic deletion of either *Gsk-3*α or *Gsk-3*β alone is insufficient to activate Wnt signaling (20–25). Only upon the deletion of three of the four total alleles for Gsk-3α and Gsk-3β is Wnt pathway activation achieved (23). Double knockout of both *Gsk-3*α and *Gsk-3*β in mouse embryonic stem cells (ESCs) (23) or mouse neural progenitors (26) results in the constitutive activation of the Wnt pathway. Importantly, while Gsk-3 regulates both insulin and Wnt signaling, these pathways do not activate one another; Wnt signaling does not activate insulin signaling and insulin signaling does not activate Wnt signaling (27–29).

Lithium is a direct inhibitor of Gsk-3 activity (30, 31) and is used therapeutically for the treatment of bipolar disorder (32). The inhibition of Gsk-3 activity has also been shown to be a potential means to reduce the pathology associated with Alzheimer’s disease (33–38). Because the dysregulation of Gsk-3 activity is believed to be an important contributor to many diseases, including Alzheimer’s disease, cancer, mental illness, and diabetes, the acquisition of more fundamental knowledge about the effects of reduced Gsk-3 activity could be important in the context of these diseases.

In this paper, we describe the analysis of genome-wide gene expression patterns in *Gsk-3*α^−^*^/^*^−^; *Gsk-3*β^−^*^/^*^−^ESCs. Since the loss of Gsk-3 activity results in the activation of numerous signaling pathways simultaneously, we further dissected the effects of losing Gsk-3 activity by examining gene expression patterns in ESCs with either constitutively active PI3K-mediated insulin signaling or constitutively active Wnt signaling.

## Materials and Methods

### Mouse embryonic stem cell culture

wild-type (WT) mouse ESCs (E14K), *Gsk-3*α^−^*^/^*^−^; *Gsk-3*β^−^*^/^*^−^(23), p110* (9), and β-catenin S33A (39) stable cell lines, along with respective control WT ESCs stably transfected with puromycin-resistant plasmids, were grown on 0.1% gelatin-coated plates in high glucose DMEM (Life Technologies) supplemented with 15% fetal bovine serum (Hyclone), 1% non-essential amino acids, 1% sodium pyruvate, 1% l-glutamine, 1% penicillin/streptomycin (Life Technologies), 55 μM 2-mercaptomethanol, and 1000 U/ml ESGRO (Millipore). Media was replenished every other day.

### RNA isolation, cDNA synthesis, and quantitative PCR

RNA was isolated from 5 × 10^5^ to 1 × 10^6^ ESCs using the MirVana Total RNA Isolation Kit (Applied Biosystems) according to the manufacturer’s instructions. Two micrograms of RNA were then used for cDNA synthesis using the high capacity cDNA reverse transcription kit (Applied Biosystems) following the manufacturer’s protocol. Quantitative RT-PCR was performed on an Applied Biosystems StepOne machine using TaqMan master mix and one of the following TaqMan assays (Applied Biosystems): Axin2 (Mm00443610_m1), Brachyury (Mm00436877_m1), Bhmt1 (Mm04210521_g1), Bhmt2 (Mm00517726_m1), Cdx2 (Mm01212280_m1), Ido2 (Mm00524206_m1), Anxa8 (Mm00507926_m1), Aqp8 (Mm0043 1846_m1), Gata6 (Mm00802636_m1), Wnt6 (Mm00437351_m1), Acta2 (Mm01546133_m1), or Foxq1 (Mm01157333_s1). Three biological replicates and three technical replicates were used for each cell type. All threshold cycle (Ct) values were normalized to a mouse Gapdh endogenous control (Mm99999915_g1) (Applied Biosystems), and relative quantification was calculated from the median Ct value (40). *P*-values were calculated using Data Assist software (Applied Biosystems).

### Microarray analysis

RNA was isolated using the MirVana Total RNA Isolation Kit (Applied Biosystems) according to the manufacturer’s instructions. The concentration of RNA was measured in a Nanodrop ND-1000 UV-Vis spectrophotometer (Nanodrop) and an Agilent 2100 Bioanalyzer Lab-On-A-Chip Agilent 6000 Series II chip (Agilent) was used to determine the integrity of the samples. RNA from each cell line was hybridized onto a SurePrint G3 Mouse GE 8 × 60 K Microarray, 8 × 60 K, AMADID 028005 (Agilent). Hybridization was performed overnight at 45°C. We performed arrays for each cell line using RNA that was isolated in biological triplicate (*n* = 3). For Gsk-3 DKO ESCs, we used *n* = 4 biological replicates. SurePrint arrays were scanned with an Agilent G2505C Microarray Scanner (Agilent). The information about each probe on the arrays was extracted from the image data using Agilent Feature Extraction 10.9 (FE) and .txt files were generated. The raw intensity values from these files in imported into the mathematical software package “R,” which was used for all data input, diagnostic plots, normalization, and quality checking steps of the analysis process using scripts developed by Dr. Peter White, Director of the Biomedical Genomics Core at Nationwide Children’s Hospital specifically for this analysis. These scripts call on several Bioconductor packages, an open-source and open-development software project to provide tools for the analysis and comprehension of genomic data (41). The algorithm used for normalization of gene expression was designed for use with Agilent One-Color Analysis. The median green (Cy3) intensities are normalized between the arrays using the Quantile Normalization package in “R” (42). Quantile normalization is a non-linear probe-level normalization that results in the same empirical distribution of intensities for each array. This is a significantly more robust approach than simply normalizing to the median value of each array. The genes that were altered by twofold either way and had a false discovery rate (FDR) of <10% were sorted and used for further interpretation of the microarray data.

### Statistical analysis of microarray data

Statistical analyses were performed using two well-validated and commonly used approaches – significance analysis of microarrays (SAM) and adjusted *p*-value. SAM is a powerful tool for analyzing microarray gene expression data useful for identifying differentially expressed genes between two conditions (43). SAM calculates a test statistic for relative difference in gene 5 expression based on permutation analysis of expression data and calculates a FDR using the *q*-value method presented in Ref. (44). In outline, SAM identifies statistically significant genes by carrying out gene specific *t*-tests and computes a statistic for each gene, which measures the strength of the relationship between gene expression and a response variable. This analysis uses non-parametric statistics, since the data may not follow a normal distribution. The response variable describes and groups the data based on experimental conditions. In this method, repeated permutations of the data are used to determine if the expression of any gene is significant related to the response. The use of permutation-based analysis accounts for correlations in genes and avoids parametric assumptions about the distribution of individual genes. For this experiment, SAM analysis was implemented in R using the Bioconductor Siggenes package. In a one-color experimental design, a *two-class unpaired* analysis is typically performed for each experimental comparison, whereas in a two-color approach a *one-class* analysis is used. Typically, an FDR cutoff in the range of 10–20% is chosen to maximize sensitivity without significantly impacting accuracy. For the current study, a 10% FDR was used to generate the list of significantly differentially expressed genes.

#### Adjusted *p*-value

The moderated *t*-statistic (*t*) is computer for each probe and for each contrast in the experimental design. This has the same interpretation as an ordinary *t*-statistic except that the standard errors have been moderated across genes, i.e., shrunk toward a common value. This has the effect of borrowing information from the ensemble of genes to aid with inference about each individual gene. The *p*-value is obtained from the distribution of the moderated *t*-statistic. Finally these *p*-values are adjusted for multiple testing using the Benjamini and Hochberg’s (45) step-up method for controlling the FDR. This is the most popular method for *p*-value adjustment. If all genes with *p*-value below a threshold, say 0.05, are selected as differentially expressed, then the expected proportion of false discoveries in the selected group is controlled to be less than the threshold value, in this case 5%. For the current study, the adjusted *p*-values were calculated using the Bioconductor limma package.

### Gene enrichment analysis

After filtering gene lists to remove duplicates, the HGNC symbols for genes increased or decreased by twofold or more were entered into ToppFun, within the ToppGene Suite (toppgene.cchmc.org) (46). ToppGene then performs functional enrichments, looking for sets of co-regulated genes. Calculations are performed using FDR for correction, and a *p*-value cutoff of <0.05.

## Results

We had previously analyzed the genome-wide expression in *Gsk-3*α^−^*^/^*^−^; *Gsk-3*β^−^*^/^*^−^ESCs (hereafter referred to as *Gsk-3* double knockout; *Gsk-3* DKO) using Affymetrix microarrays, and found hundreds of genes whose expression was significantly increased or decreased compared to WT ESCs (9). Here, we repeated this microarray analysis using the Agilent platform. For all of the microarray gene expression analyses, we used a twofold or greater change in gene expression as our threshold for significance. We also isolated RNA and performed the hybridization to the microarrays at the same time to reduce experiment-to-experiment variability. Consistent with our previous results using the Affymetrix platform, we found 1313 genes up-regulated twofold or more in *Gsk-3* DKO ESCs, while 2178 genes were down-regulated twofold or more (a complete list of genes can be found in Datasheet 1 in Supplementary Material). One of the important signaling pathways regulated by Gsk-3 is the Wnt pathway (47). We therefore expected our microarray data from *Gsk-3* DKO ESCs to reveal many of the same Wnt target genes that have been demonstrated experimentally in a variety of model systems [Wnt Homepage, www.stanford.edu/group/nusselab/cgi-bin/wnt/target_genes]. A few well-established direct targets of Wnt signaling, such as *Brachyury* (*T*) and *Axin2*, were found to be increased substantially in *Gsk-3* DKO ESCs (162.5- and 8.6-fold, respectively) (Table 1). We were surprised, however, to find that *Brachyury* and *Axin2* were the exception of the 65 Wnt putative target genes; we identified in our microarray data from *Gsk-3* DKO ESCs that only seven additional genes (*Sp5, Cdx1, Stra6, Lef1, Cyclin D1, PTTG*, and *Fgf18*) had their expression increased more than twofold (Table 1). In fact, 13 Wnt target genes showed decreased expression of more than twofold (Table 1).

**Table 1:** **Comparison of known Wnt target genes to their expression levels in Gsk-3 DKO and S33A ESCs as determined by microarray**.

Gene	DKO	S33A
Brachyury	162.5	41.7
SP5	75.8	15.1
Cdx1	16.5	82.5
Axin-2	8.6	4.6
Stra6	4.8	1.3
LEF1	4	−7
Cyclin D	3.1	−3.5
Pituitary tumor transforming gene (PTTG)	2.2	1.8
FGF18	2.1	−2.9
BMP4	1.8	1.2
PPARδ	1.6	1.1
Endothelin-1	1.5	1.4
Telomerase	1.5	1.4
LGR5/GPR49	1.5	−1.2
Jagged	1.4	1
sFRP-2	1.4	−1.5
CD44	1.3	1.4
c-myc binding protein	1.3	−1.9
Msl1	1.3	1.2
Nitric oxide synthase 2	1.3	1.4
Nkx2.2	1.3	1.2
c-myc	1.2	−3.3
Met	1.2	−1.2
Claudin-1	1.1	1
Id2	1.1	1.2
FoxN1	1.1	1.1
Gbx2	1.1	−2.2
MMP-7	1	1
Osteoprotegerin	1	1
Wnt3a	1	1.2
Neurogenin 1	1	1.1
Tiam1	−1.1	−1.2
FGF20	−1.1	−1.4
Cdx4	−1.1	2.3
Sox17	−1.2	−1.6
Oct-4	−1.2	−1.3
Runx2	−1.3	1
Sox2	−1.3	1.4
Frizzled 7	−1.3	−2.1
Gastrin	−1.4	1.3
Sox9	−1.4	−1.1
CCN1/Cyr61	−1.4	−1.2
Follistatin	−1.4	1.2
NeuroD1	−1.5	−5.6
c-jun	−1.6	−2.2
Nr-CAM	−1.6	−1.5
SALL4	−1.6	−2.3
Nanog	−1.6	1.4
FGF9	−1.7	1
Irx3 and Six3	−1.8	−1.4
Cacna1γ	−1.8	−1.8
MMP2, MMP9	−1.9	1.6
LBH	−2	1.4
Pitx2	−2.1	− 1.5
ITF-2	−2.3	− 1.9
Twist	−2.4	−2
Periostin	−2.4	1
VEGF	−2.5	2.4
Snail	−2.8	− 1.5
Tcf-1 (Hnf1a)	−3	− 1.1
n-myc	−3.4	−3.4
Gremlin	−3.5	−3.5
EGF receptor	−3.8	− 1.5
Delta-like 1	−9.8	−4.3
WISP	−10	− 1.1

*The list of genes was found at The Wnt Homepage website (www.stanford.edu/group/nusselab/cgi-bin/wnt/target_genes). Genes whose expression increased more than twofold are shown in red, while genes whose expression decreased more than twofold are shown in green*.

The relative paucity of up-regulated Wnt target genes was surprising, especially, since it has been shown that a reporter construct containing multimerized Lef/Tcf binding sites is strongly activated in *Gsk-3* DKO ESCs [(23); unpublished observation]. Therefore, we wanted to compare the gene expression data from *Gsk-3* DKO ESCs with another mouse embryonic stem cell line that has constitutively active Wnt signaling. WT ESCs stably expressing a form of β-catenin in which serine 33 has been mutated to an alanine (S33A) that prevents the phosphorylation by Gsk-3 on this residue as well as subsequent ubiquitination, resulting in the constitutive activation of Wnt signaling. These cells have previously been shown to potently activate the expression of several Wnt target genes (39). The β-catenin S33A cells and their control cells were included in our microarray experiment to investigate how similar the patterns of gene expression were compared to *Gsk-3* DKO ESCs.

Microarray data showed that 1468 genes were up-regulated twofold or more in S33A cells compared to control cells, while 1412 genes were down-regulated twofold or more. As expected, we confirmed the high levels of *Brachyury, Axin2*, and *Cdx1* that were also observed in the *Gsk-3* DKO ESCs; however, only three additional genes (*Sp5, Cdx4*, and *VEGF*) showed a twofold or greater increase in gene expression in the S33A ESCs (Table 1). And similar to the data from *Gsk-3* DKO ESCs, 12 Wnt target genes are down-regulated twofold or more in S33A cells (Table 1). Taken together, these data show that some Wnt target genes, such as *Brachyury* and *Axin2*, are indeed transcribed at high levels in two different cell lines in which Wnt signaling is constitutively active – *Gsk-3* DKO and β-catenin S33A. Our data also show that many Wnt target genes are not activated in these cell lines as was predicted.

Initially, we had expected that many of the genes whose expression was significantly increased in *Gsk-3* DKO ESCs would be due to activation of the Wnt pathway. Since we did not observe the increased expression of Wnt target genes, we asked whether the activation of another Gsk-3-dependent signaling pathway could explain the data. Because the genetic deletion of Gsk-3 isoforms likely leads to the activation of signaling pathways other than the Wnt pathway, we speculated that the combined effect of multiple pathway activation likely leads to new patterns of gene expression. Another important signal transduction pathway that is regulated by Gsk-3 activity is the insulin signaling pathway. To investigate the contribution of insulin signaling to the effects on gene expression seen in the *Gsk-3* DKO ESCs, we analyzed genome-wide microarray gene expression data from WT ESCs stably expressing a constitutively active form of the p110 subunit of PI3K (p110*) (9, 10). Eight hundred sixty-four genes were up-regulated twofold or more in p110* ESCs compared to control cells, while 1660 genes were down-regulated twofold or more.

**Figure 1:**
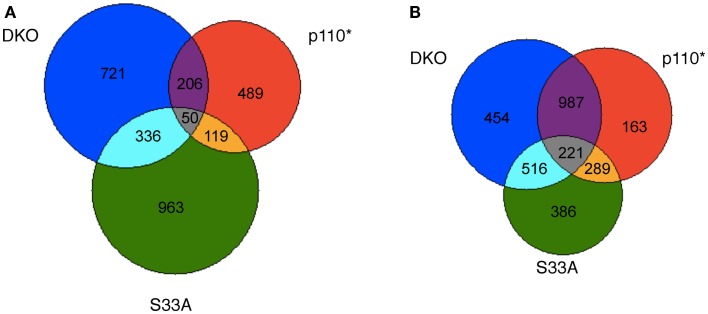
Venn diagram representing the overlap in genes whose expression is changed at least twofold in *Gsk-3* DKO, p110*, and β-catenin S33A ESCs. **(A)** Representation of genes whose expression was increased in *Gsk-3* DKO (blue; 1313 genes total), p110* (red; 864 genes total), and S33A ESCs (green; 1468 genes total). Genes whose expression is increased in multiple genes sets are denoted by the numbers at the interface of the gene sets. **(B)** Representation of genes whose expression was decreased in *Gsk-3* DKO (blue; 2178 genes total), p110* (red; 1660 genes total), and S33A ESCs (green; 1412 genes total). Genes whose expression is decreased in multiple genes sets are denoted by the numbers at the interface of the gene sets.

After compiling the list of genes whose expression was significantly changed in *Gsk-3* DKO, p110*, and S33A ESCs, we were curious as to the similarities and disparities in the patterns of gene expression in these cell lines. To perform this evaluation, we removed all duplicate probes from our lists of genes from the microarray experiment, and we then sorted based on changes seen in each list. The *Gsk-3* DKO and p110* ESCs had 1313 and 864 genes, respectively, that were up-regulated twofold or more. Of these, 206 genes were up-regulated in both cell types (Figure 1A). Similarly, 1468 genes were up-regulated by at least twofold in S33A cells, and 336 of these genes overlapped with the 1313 genes up-regulated in the *Gsk-3* DKO ESCs (Figure 1A). As we expected, there was relatively little overlap in the genes up-regulated in both p110* and S33A ESCs; only 119 genes were in common between these gene sets (Figure 1A). This comparison of up-regulated genes shows a greater degree of overlap between *Gsk-3* DKO and S33A ESCs than with *Gsk-3* DKO and p110* ESCs.

Interestingly, the similarities between gene sets were even greater when examining the genes whose expression was down-regulated twofold or more. Two thousand one hundred seventy-eight genes showed decreased expression in *Gsk-3* DKO ESCs, compared with 1660 genes in p110* ESCs and 1412 genes in S33A ESCs. Furthermore, we found that 987 genes were in common between p110* and *Gsk-3* DKO ESCs, while only 516 genes were shared between S33A and *Gsk-3* DKO ESCs (Figure 1B). This pattern is striking in that it is the opposite of what was seen with the up-regulated genes, with a greater overlap between *Gsk-3* DKO and p110* ESCs. In addition, the overlap between p110* and S33A ESCs was also more pronounced, with the same 289 genes down-regulated at least twofold in both cell lines (Figure 1B). These data suggest that the loss of *Gsk-3* in ESCs results in the up-regulation of mostly distinct genes in either p110* or S33A ESCs, but that those genes that are down-regulated in the absence of *Gsk-3* share a greater overlap in cells with activated PI3K signaling and activated Wnt signaling.

Since different sets of genes were either increased or decreased in expression in p110* and S33A ESCs, we bioinformatically performed enrichment analyses to see if functionally related genes were co-regulated in a signaling pathway-specific fashion (Tables 2 and 3). Genes that were up-regulated in *Gsk-3* DKO ESCs were involved in gene ontology (GO): molecular function of protein domain (GO:0019904) and also the GO: biological process for cilium organization (GO:0044782). In addition, genes encoding for CD molecules, tumor necrosis factor receptor superfamily, and intraflagellar transport homologs were up-regulated. Genes that were increased in expression in p110* ESCs showed an enrichment for the GO: molecular function of piRNA binding (GO:0034584) and an showed an increase in expression in genes encoding for zinc fingers, C2H2, and BTB domain-containing (ZBTB). In addition, both *Gsk-3* DKO and p110* ESCs showed increased expression of genes involved in urothelium and lower urinary tract development. For genes up-regulated in S33A ESCs, the top GO: molecular function clusters were genes involved in organic acid binding (GO:0043177) and protein homodimerization activity (GO:0042803). Also, a significant number of genes that contain homeobox domains were up-regulated in the S33A ESCs, as well as a high number of genes encoding for CD molecules.

**Table 2:** Summary of enrichment analysis of genes up-regulated in Gsk-3 DKO, p110*, and S33A ESCs.

Name	ID	*p*-Value	Hit count
**GSK-3 DKO ESCs**			
**GO: molecular function**
Protein domain specific binding	GO:0019904	1.01 × 10^−5^	55/633
**GO: biological process**			
Cilium organization	GO:0044782	6.06 × 10^−6^	19/123
**Gene family**			
CD molecules	CD	5.14 × 10^−8^	21/276
Tumor necrosis factor receptor superfamily	TNFRSF	5.77 × 10^−5^	4/12
Intraflagellar transport homologs	IFT	1.52 × 10^−4^	4/15
**Coexpression Atlas**			
Mendel_RNAseq_e17.5_urothelium		2.74 × 10^−16^	101/989
Developing lower urinary tract_e13.5		1.04 × 10^−13^	82/802
P110* **ESCs**			
**GO: molecular function**			
piRNA binding	GO:0034584	3.17 × 10^−5^	3/3
**Gene family**			
Zinc fingers, C2H2, and BTB domain containing	ZBTB	5.33 × 10^−5^	6/47
**Coexpression Atlas**			
Mendel_RNAseq_e17.5_urothelium		1.58 × 10^−10^	65/989
Developing lower urinary tract_e13.5		1.30 × 10^−6^	30/802
**S33A ESCs**			
**GO: molecular function**			
Organic acid binding	GO:0043177	4.93 × 10^−5^	26/230
Protein homodimerization activity	GO:0042803	5.15 × 10^−5^	56/674
**Gene family**			
CD molecules	CD	5.08 × 10^−5^	18/276

*Bonferroni correction method*.

*All *p*-Value cutoffs – 0.05*.

**Table 3:** Summary of enrichment analysis of genes down-regulated in Gsk-3 DKO, p110*, and S33A ESCs.

Name	ID	*p*-Value	Hit count
**GSK-3 DKO ESCs**			
**GO: molecular function**			
Sequence-specific DNA binding	GO:0043565	8.59 × 10^−13^	109/726
Sequence-specific DNA binding transcription factor activity	GO:0003700	7.28 × 10^−12^	141/1067
**GO: biological process**			
Cardiovascular system development	GO:00722358	2.15 × 10^−21^	148/871
**Pathway**			
Extracellular matrix organization		1.40 × 10^−9^	50/264
Elastic fiber formation		1.51 × 10^−7^	15/41
P110* **ESCs**			
**GO: molecular function**			
Receptor binding	GO:0005102	1.51 × 10^−11^	133/1341
**GO: biological process**			
Organ morphogenesis	GO:0009887	2.09 × 10^−34^	147/876
Extracellular matrix organization	GO:0030198	1.59 × 10^−22^	70/330
Cardiovascular system development	GO:0072358	2.34 × 10^−22^	124/871
**Pathway**			
Extracellular matrix organization		6.17 × 10^−16^	54/264
**S33A ESCs**			
**GO: molecular function**			
Sequence-specific DNA binding	GO:0043565	2.15 × 10^−10^	81/726
Sequence-specific DNA binding transcription factor activity	GO:0003700	3.54 × 10^−10^	105/1067
**GO: biological process**			
Neurogenesis	GO:0022008	3.88 × 10^−15^	143/1360
Extracellular matrix organization	GO:0072358	1.21 × 10^−14^	104/871

*Bonferroni correction method*.

*All *p*-Value cutoffs – 0.05*.

We found it notable that the enrichments among down-regulated genes were more robust than among the up-regulated genes (Table 4). For genes decreased twofold or more in *Gsk-3* DKO ESCs, the greatest enrichment for GO: molecular function was in genes encoding for sequence-specific DNA binding, i.e., transcription factors (GO:0043565). In addition, a large number of genes involved in the development of the cardiovascular system (GO:0072358) were down-regulated in *Gsk-3* DKO ESCs. Also decreased in expression were genes involved in extracellular matrix organization and elastic fiber formation. For down-regulated genes in p110* ESCs, the most enriched GO: molecular function was for genes involved in receptor binding (GO:0005102), while the highest enrichment for GO: biological processes were for organ morphogenesis (GO: 0009887), extracellular matrix organization (GO:0030198), and cardiovascular system development (GO:0072358). Furthermore, many genes involved in extracellular matrix organization were decreased in p110* ESCs. Genes down-regulated in S33A ESCs were enriched for genes encoding sequence-specific DNA binding (GO:0043565), as well as enrichment for genes involved in neurogenesis (GO:0022008) and cardiovascular system development (GO:0072358). The results from these enrichment analyses showed several similarities and differences with respect to the genes that have increased or decreased expression in *Gsk-3* DKO, p110*, and S33A ESCs, and provide a framework for beginning to better understand the complex interrelationships between PI3K-mediated signaling and Wnt signaling, as well as providing important insights into the patterns of gene expression seen in *Gsk-3* DKO ESCs.

**Table 4:** Summary of comparison between microarray and qPCR gene expression data in Gsk-3 DKO, S33A, and p110* ESCs for genes described in this work.

	DKO		S33A		p110*	
	Microarray	qPCR	Microarray	qPCR	Microarray	qPCR
Axin2	6.7×	7.0×	3.6×	3.7×	1.7×	−2.5×
Brachyury	162.5×	622×	41.7×	38×	−1.5×	−1.4×
Bhmt2	5.7×	13.1×	60.8×	56.5×	−2.6×	1.3×
Cd×2	27.6	5.3×	33.3×	12.5×	1.2×	1×
Bhmt1	6.5×	14.1×	49.1×	70.1×	−5.5×	2.8×
Ido2	9.4×	81.4×	3.4×	20×	1.3×	1.4×
Wnt6	2.3×	1.2×	1.5×	−1.4×	10.1×	5.7×
An×a8	1.5×	1.7×	1.5×	1.4×	6.8×	3.6×
Gata6	−1.4×	−1.1×	−3.1	−2.5×	−31.8×	3.4×
Aqp8	−1.5×	1.6×	−2.6	−3.3×	−48.9×	1.7×
Fo×q1	−1.4×	−1.1×	−2.9×	1.5×	−31.5×	−1.7×
Acta2	−400.3×	−5.0×	−1.8×	2.3×	−165.6×	−2.5×

*Decreases in gene expression are denoted by negative values*.

**Figure 2:**
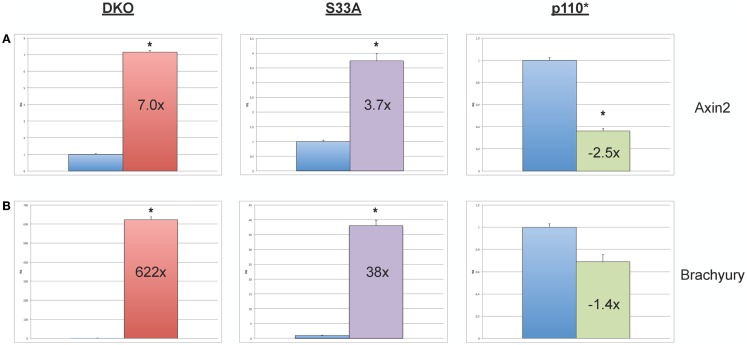
Validation of the expression of the Wnt target genes, *Axin2* and *Brachyury* in *Gsk-3* DKO ESCs, β-catenin S33A ESCs, and p110* ESCs. **(A)** Quantitative PCR of *Axin2* showing the expression, relative to WT control. **(B)** Quantitative PCR of *Brachyury* showing the expression, relative to WT control. For both results: RQ = relative quantification (all values were normalized to a *Gapdh* endogenous control). Error bars represent standard error of the mean (SEM) between biological replicates (*n* = 3) and technical replicates (*n* = 3; *n* = 9 total for each gene in each cell line) (*p < 0.001, two-tailed *t*-test). Fold changes compared to WT are shown.

While the data obtained from microarray experiments can provide an excellent overall view of differential patterns of gene expression, it is nonetheless very important to use an independent technique to experimentally validate the changes in gene expression that were observed via microarray. Therefore, we selected a handful of genes whose expression patterns we of interest, and we performed quantitative reverse-transcriptase PCR (qPCR) using TaqMan probes on RNA isolated from *Gsk-3* DKO, p110*, S33A ESCs, and their respective control cell lines. As an initial quality control measure, we performed qPCR to validate the expression of the known Wnt target genes, *Axin2* and *Brachyury*. As expected, we saw substantial increases in gene expression in both *Gsk-3* DKO and S33A ESCs for *Axin2* (up 7-fold in *Gsk-3* DKO and 3.7-fold in S33A ESCs) and *Brachyury* (up 622-fold in *Gsk-3* DKO and 38-fold in S33A ESCs), while both genes has reduced expression in p110* ESCs (0.4- and 0.7-fold changes, respectively) (Figure 2). These results are consistent with *Axin2* and *Brachyury* being Wnt target genes.

**Figure 3:**
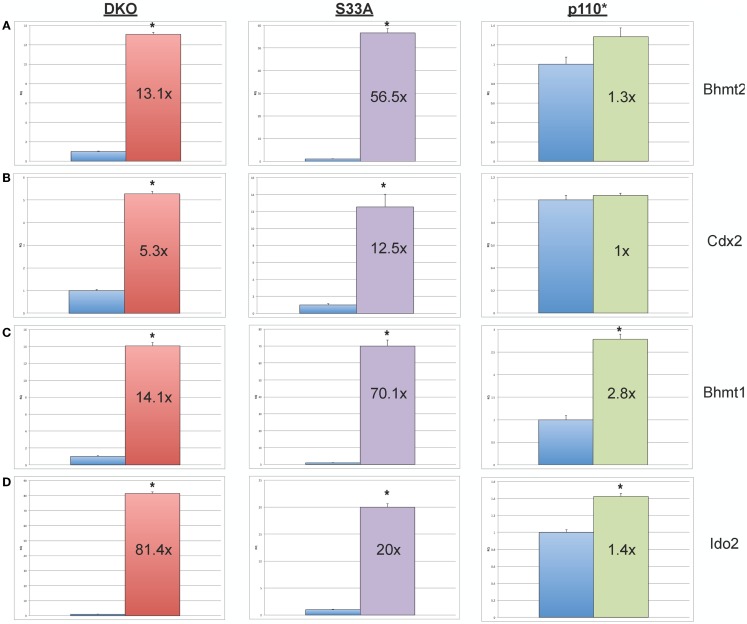
Validation of genes whose expression is increased in *Gsk-3* DKO and β-catenin S33A ESCs, but not p110* ESCs. **(A)** qPCR of *Bhmt2* showing the expression, relative to WT control. **(B)** qPCR of *Cdx2* showing the expression, relative to WT control. **(C)** qPCR of *Bhmt1* showing the expression, relative to WT control. **(D)** qPCR of *Ido2* showing the expression, relative to WT control. For all results shown: RQ = relative quantification (all values were normalized to a *Gapdh* endogenous control); error bars represent standard error of the mean (SEM) between biological replicates (*n* = 3) and technical replicates (*n* = 3; *n* = 9 total for each gene in each cell line) (*p < 0.001, two-tailed *t*-test); and fold changes compared to WT are shown.

We then proceeded to validating the expression of additional genes whose expression levels were shown to be substantially increased or reduced in the microarray experiments. We selected four genes, *Bhmt1* (*Betaine-homocysteine methyltransferase*), *Bhmt2* (*Betaine-homocysteine methyltransferase* 2), *Cdx2* (*Caudal-type homeobox 2*), and *Ido2* (*Indoleamine 2,3-dioxygenase 2*), whose expression, by microarray, was shown to be up-regulated in both *Gsk-3* DKO and S33A ESCs, but not p110* ESCs. Validation by qPCR showed that the expression of *Bhmt1* was up 14.1- and 70.1-fold in *Gsk-3* DKO and S33A ESCs, respectively, compared to a modest 2.8-fold increase in p110* ESCs (Figure 3). Similarly *Bhmt2* expression was shown to be increased 13.1-fold in *Gsk-3* DKO ESCs and 56.5-fold in S33A ESCs, while expression was only increased by 1.3-fold in p110* ESCs (Figure 3). The expression of *Cdx2* was increased 5.3- and 12.5-fold in *Gsk-3* DKO ESCs and S33A ESCs, respectively, but remained unchanged in p110* ESCs (Figure 3). Finally, qPCR showed that *Ido2* expression was increased 81.4-fold in *Gsk-3* DKO ESCs and 20-fold in S33A ESCs, while showing a minimal 1.4-fold increase in expression in p110* ESCs (Figure 3).

**Figure 4:**
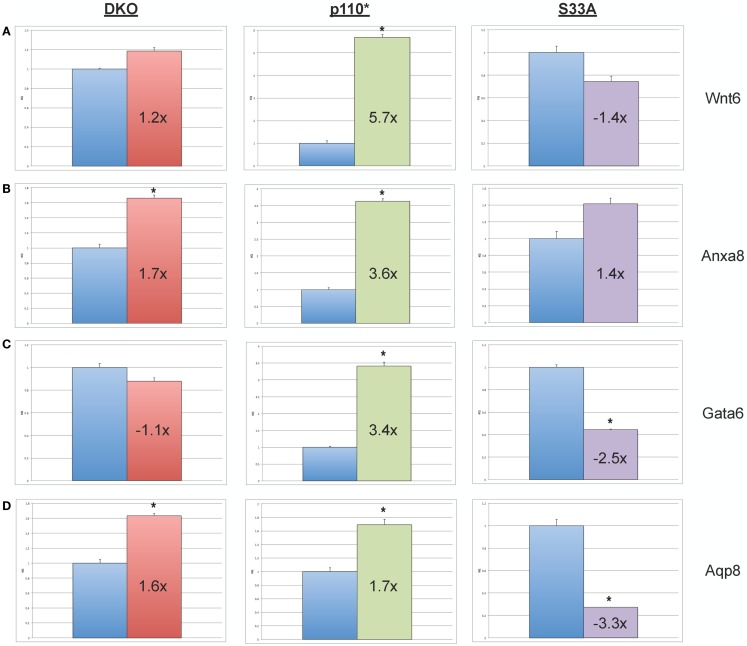
Validation of genes whose expression is increased in p110* ESCs, but not S33A ESCs. **(A)** qPCR of *Wnt6*. **(B)** qPCR of *Anxa8*. **(C)** qPCR of *Wnt6*. **(D)** qPCR of *Anxa8*. For all results shown: RQ = relative quantification (all values were normalized to a *Gapdh* endogenous control); error bars represent standard error of the mean (SEM) between biological replicates (*n* = 3) and technical replicates (*n* = 3; *n* = 9 total for each gene in each cell line) (*p < 0.001, two-tailed *t* test); and fold changes compared to WT are shown.

**Figure 5:**
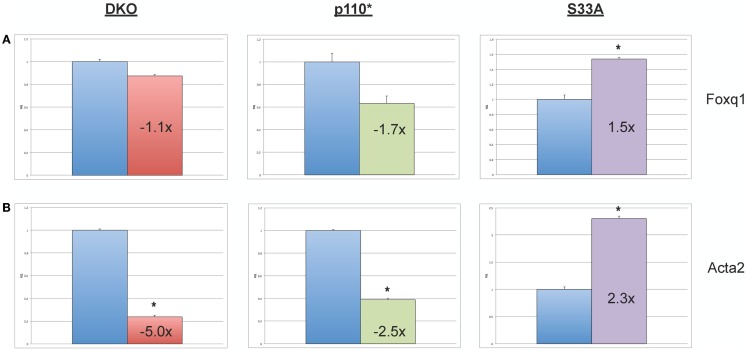
Validation of genes whose expression is decreased in p110* ESCs, but not S33A ESCs. **(A)** qPCR of *Foxq1*. **(B)** qPCR of *Acta2*. For both results: RQ = relative quantification (all values were normalized to a *Gapdh* endogenous control); error bars represent standard error of the mean (SEM) between biological replicates (*n* = 3) and technical replicates (*n* = 3; *n* = 9 total for each gene in each cell line) (*p < 0.001, two-tailed *t* test); and fold changes compared to WT are shown.

Next, we chose to perform similar qPCR validation on genes whose expression was changed in *Gsk-3* DKO and p110* ESCs, but not in S33A ESCs. We selected six genes for analysis – *Wnt6, Anxa8 (Annexin 8), Gata6 (GATA binding protein 6), Aqp8 (Aquaporin 8), Foxq1 (Forkhead box Q1)*, and *Acta2 (actin*, α*2, smooth muscle, aorta)*. All of the genes that we assayed showed lower changes in gene expression compared to that seen by microarray. For example, microarray data showed that *Wnt6* and *Anxa8* were up-regulated in p110* ESCs 10.1- and 6.8-fold, respectively; however, qPCR revealed that the expression of *Wnt6* and *Anxa8* was about half of that – 5.7- and 3.6-fold, respectively (Figure 4). Similarly, while *Gata6, Aqp8, Foxq1*, and *Acta2* all showed at least 30-fold reductions in gene expression in p110* ESCs by microarray, the qPCR results revealed more subtle changes, ranging from a 3.4-fold increase for *Gata6* to a 2.5-fold reduction in *Acta2* (Table 3). Importantly, the expression of these genes in S33A ESCs was quite different. For example, while *Gata6* expression was up 3.4-fold in p110* ESCs, *Gata6* expression was decreased 2.5-fold in S33A ESCs (Figure 5). Similarly, *Acta2* expression was decreased 2.5-fold in p110* ESCs, while *Acta2* expression was increased 2.3-fold in S33A ESCs (Figure 5). These results are consistent with opposing effects of PI3K-mediated insulin signaling and Wnt signaling having distinct effects on gene expression.

## Discussion

The analysis of gene expression changes in *Gsk-3* DKO ESCs revealed almost 3500 genes whose expression increased or decreased at least twofold, reinforcing the notion that Gsk-3 activity broadly regulates the expression of many genes. Our data, which examined global changes in gene expression in several clonal cell lines, illuminated numerous interesting and unexpected features of Gsk-3-dependent signal transduction.

We had initially expected to find many known Wnt target genes to be up-regulated in a Gsk-3 and β-catenin-dependent fashion and this was indeed true for *Axin2* (48) and *Brachyury*. However, most of the other Wnt target genes did not show significant increases in expression, and in fact, almost twice as many genes showed decreases in expression. It is possible that this simply reflects differences in the regulation of gene expression between cell types; alternatively, this data could provide a readout of affected genes due to the constitutive activation of Wnt signaling arising from the genetic deletion of *Gsk-3*α and *Gsk-3*β. Furthermore, since these ESCs have been deficient in *Gsk-3*α and *Gsk-3*β since their successful homologous recombination event, it is possible that downstream negative feedback effects serve to modify the expression of Wnt target genes, making the changes in gene expression not appear as dramatic as expected.

We do, however, identify several putative novel Wnt target genes, whose expression is increased in both *Gsk-3* DKO and β-catenin S33A ESCs. *Cdx2, Ido2, Bhmt1*, and *Bhmt2* were all confirmed by qPCR to conform to the microarray expression profiling. Interestingly, the transcription factor *Cdx2*, was recently shown to be a direct target of Wnt signaling in the mouse, independently confirming our observation (49). Furthermore, while *Ido1* has been shown to be a Wnt target gene (50), *Ido2* has not. In the mouse genome, the *Ido1* and *Ido2* genes lie next to each other on chromosome 8 (8qA2) and are transcribed in the same direction. Thus, one possibility is that β-catenin-mediated transcription could influence both genes from the *Ido1* promoter. Alternatively, there may be additional Lef/Tcf binding sites in the *Ido2* promoter. An *in silico* search (51, 52) for Lef/Tcf binding sites in the mouse *Ido2* promoter identified eight putative binding sites within 2 kb of the transcription start site (TSS). Further detailed studies will be required to verify whether any of these putative binding sites are bona fide Lef/Tcf binding sites.

Similarly, Bhmt1 and Bhmt2 are also contiguous in the mouse genome (13qC3). Neither gene had previously been shown to be regulated by Wnt signaling, but we find both genes have increased expression in *Gsk-3* DKO ESCs (14.1- and 13.1-fold, respectively) and even more potently expressed in β-catenin S33A ESCs (up 70.1- and 56.5-fold, respectively). *Bhmt1* and *Bhmt2* both encode for betaine–homocysteine *S*-methyltransferase enzymes, participating in the regulation of homocysteine levels (53). Interestingly, variations in *BHMT1* and *BHMT2* have also been implicated in the development of cleft palate in certain human populations (54–56). Interestingly, mice deficient in *Gsk-3*β develop cleft palate (21, 24, 25). It is unclear whether this phenotype is a consequence of altered expression of *Bhmt1* or *Bhmt2*, but based on the data presented here, there may be a functional connection between these observations.

While not as dramatic as the changes seen for putative Wnt target genes, we found several genes whose expression was insensitive to constitutively active Wnt signaling, but were increased or decreased in expression in a Gsk-3/PI3K-dependent manner. It has been demonstrated in animal models of various cancers that the transcription factor ZEB1 activates PI3K signaling (57–59) and this results in increased expression of *Gata6* (60). Our data from mouse ESCs stably expressing a constitutively active form of the p110 subunit of PI3K corroborates these findings and show that the PI3K-mediated increase in *Gata6* expression is not limited to cancer cell populations. *Wnt6, Anxa8*, and *Aqp8* also showed increased expression in p110* ESCs, while all but *Anxa8* had reduced expression in S33A ESCs. While PI3K signaling has been shown to regulate the subcellular localization of *Aqp8* in hepatocytes (61), this is the first report that activation of PI3K signaling has an effect on *Aqp8* transcription. *Anxa8* expression has been shown to be down-regulated by epidermal growth factor (EGF)-stimulated PI3K signaling in a model of metastatic cholangiocarcinoma (62), while we find that *Anxa8* expression is increased 3.6-fold in p110* ESCs, likely reflecting cell-type differences in *Anxa8* gene regulation. It should be mentioned that we selected *Gata6, Wnt6, Anxa8*, and *Aqp8* for validation by qPCR because these genes showed increased expression in both *Gsk-3* DKO and p110* ESCs by microarray; however, the qPCR results showed only modest changes in gene expression in the *Gsk-3* DKO ESCs, highlighting the importance of not relying on microarray gene expression data alone.

Finally, we also found that the expression of *Foxq1* and *Acta2* were decreased in p110* ESCs. *Acta2* showed the most robust changes in expression (down fivefold in *Gsk-3* DKO ESCs, down 2.5-fold in p110* ESCs, and up 2.3-fold in S33A ESCs). We did find that *Foxq1* expression is modestly decreased in p110* ESCs and increased 1.5-fold in S33A ESCs. *Foxq1* has recently been shown to be one of the most highly expressed genes in human colorectal cancer and has been shown to be a direct target of Wnt signaling, which is often constitutively activated in colorectal cancer cells (63). Based on these data, we expected to find increased *Foxq1* expression in both *Gsk-3* DKO and S33A ESCs, but we did not observe these expected increases. It will be interesting to determine the mechanism responsible for the lack of response by *Foxq1* to constitutively activated Wnt signaling in the context of mouse ESCs.

Taken together, our data shows that, while many genes have their expression changed in *Gsk-3* DKO ESCs, only a fraction of the changes we observe are due to activated Wnt signaling or activated PI3K signaling, meaning that there are likely several other signaling pathways whose activation affects *Gsk-3*-dependent gene expression. Thus, our data provide a framework for future analyses to make these connections. Furthermore, while these studies were performed in mouse ESCs, we believe that the findings from this study can be used in future studies examining the role of insulin or Wnt signaling pathways in other biological settings, such as the study of insulin resistance in the diabetic brain.

## Conflict of Interest Statement

The authors declare that the research was conducted in the absence of any commercial or financial relationships that could be construed as a potential conflict of interest.

## Supplementary Material

The Supplementary Material for this article can be found online at http://www.frontiersin.org/Journal/10.3389/fendo.2014.00133/abstract

Datasheet 1 Microarray gene expression data for Gsk-3 DKO ESCs, β-catenin S33A ESCs and p110* ESCs. Tabs at the bottom of the spreadsheet contain the microarray data, either as All Data, or sorted by fold changes in expression as seen in Gsk-3 DKO, p110*, or β-catenin S33A ESCs. Within each tab is found the Agilent probe ID, Gene symbol, Gene name, NCBI accession number, Entrez Gene ID, fold-changes (FC) compared to WT ESCs, false-discovery rates (FDR) compared to WT ESCs, and adjusted P-values (AdjP) compared to WT ESCs. The normalized microarray values for each individual sample, as well as the averages, are also shown.
